# The role of the complement system in gastrointestinal-related diseases

**DOI:** 10.3389/fimmu.2026.1801628

**Published:** 2026-05-13

**Authors:** Bo Yong, Zixiang Luo, Bo Luo, Jinbo Liu, Zhangrui Zeng, Xiaoli Zheng

**Affiliations:** 1 School of Basic Medical Sciences, Southwest Medical University, Luzhou, Sichuan, China; 2 Department of Laboratory Medicine, the Affiliated Hospital of Southwest Medical University, Luzhou, China

**Keywords:** complement system, gastrointestinal-related diseases, inflammatory bowel disease, intestinal tumors, therapeutic targets

## Abstract

The complement system bridges innate and adaptive immunity, orchestrating immune surveillance and maintaining tissue homeostasis. This review summarizes the role of the complement system in gastrointestinal-related diseases, including its function in innate immunity, impact on adaptive immunity, and regulatory role under physiological and pathological conditions in the gut. The activation pathways, regulatory mechanisms, and interactions of the complement system with the gut microbiome are crucial for maintaining intestinal homeostasis. Genetic deficiencies or excessive activation of the complement system are closely associated with the development of various intestinal diseases, including infections, inflammatory bowel disease (IBD), and tumors. This review also explores the role of the complement system in the tumor microenvironment (TME) of the gut and its potential as a therapeutic target. By deepening our understanding of the mechanisms by which the complement system operates in gastrointestinal diseases, we can provide a theoretical foundation for the development of novel treatment strategies.

## 1 Introduction to complement

### 1.1 The complement system

Discovered by Jules Bordet in the late 19th century, the complement system was initially defined as a circulating serum system that complements antibody and cell-mediated immune responses. The complement system acts as a bridge between innate and adaptive immunity in anti-infectious defense mechanisms. However, genetic deficiencies, functional disorders, and excessive activation of the complement system are associated with the pathology of certain diseases (1–3).

**Figure 1:**
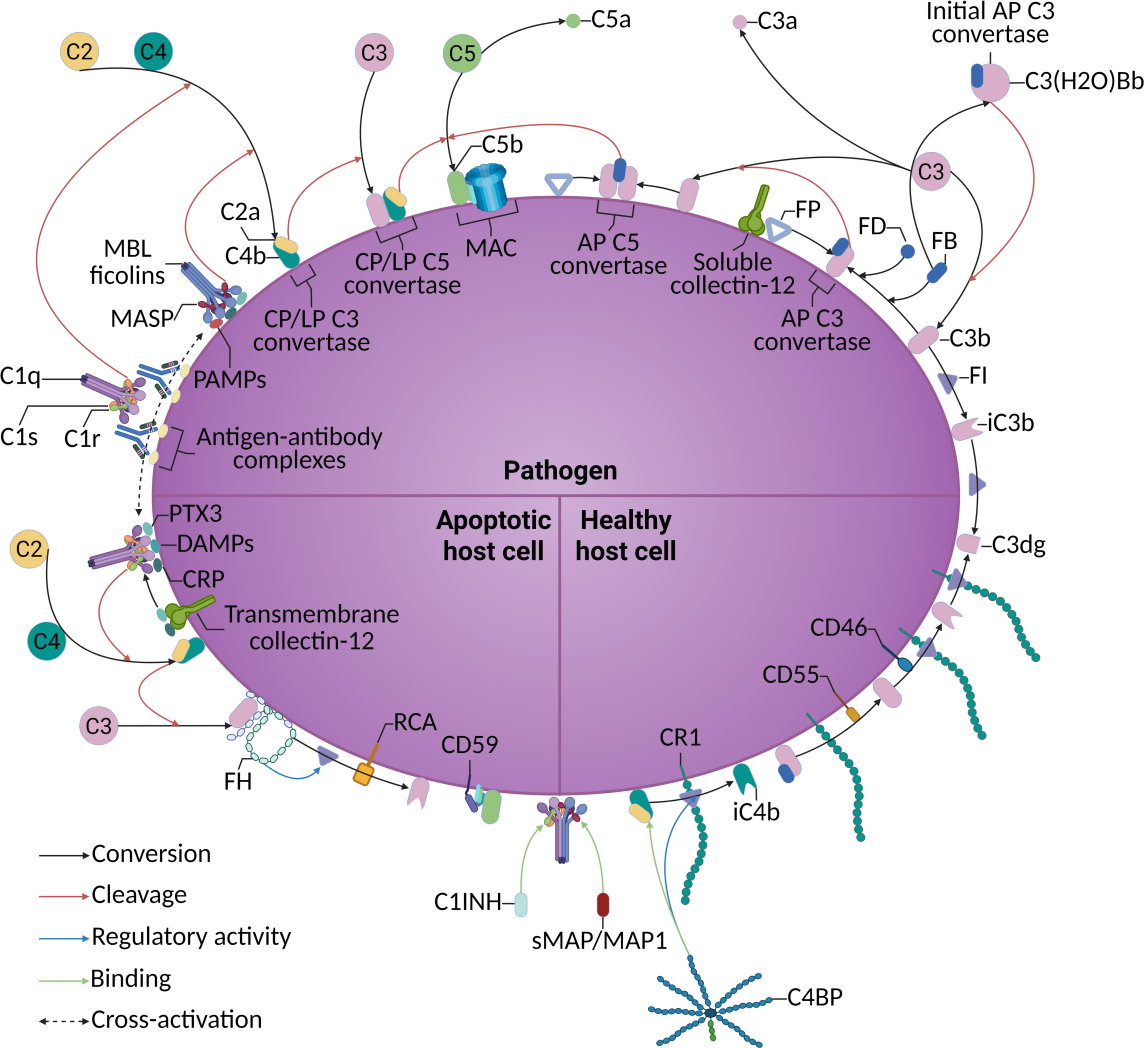
Different activation pathways and regulatory mechanisms of the complement system under physiological conditions.

The complement system is an integral part of the innate immune system, consisting of over 50 blood and lymphatic circulation proteins (primarily produced by the liver) and membrane-bound proteins. Protein components, proteases, pattern recognition molecules (PRMs), regulatory factors, and cell surface receptors are crucial for modulating the complement system’s response to different stimuli. Functionally, the complement system can be divided into two main parts: the enzymatic cascade and the lytic pathway (**Figure 1**). The primary function of the cascade is to assemble convertases, leading to the activation and amplification of the complement system. This includes three activation pathways: the classical pathway, lectin pathway, and alternative pathway. Different microbial components, antigen-antibody complexes, and other exogenous or endogenous substances can activate the complement system by initiating a series of serine protease cascades. The products of complement activation possess biological functions such as regulating phagocytosis (C3b), lysing cells (C5b-9), mediating inflammation (C3a, C5a), modulating immune responses (C3a, C5a, C3b, C3d), and clearing immune complexes (C3b, iC3b) (4, 5).

### 1.2 Complement activation pathways

The classical pathway is primarily triggered by the binding of the C1 complex, which consists of C1q and the serine proteases C1r and C1s, to immunoglobulins on the surface of pathogens (6–8). Additionally, pentraxins, a superfamily of soluble PRMs including C-reactive protein (CRP) and pentraxin 3 (PTX3), recognize and bind to pathogen surface components, which then activate C1q (9). Activation of C1q leads to conformational changes, resulting in the formation of enzymatically active C1r and C1s (10). The resulting C1 enzyme complex mediates the sequential cleavage of C4 and C2, generating the C3 convertase C4b2a (11). In the lectin pathway, pattern recognition molecules include mannose-binding lectin (MBL), ficolins, and collectins, which can directly recognize glycan structures on the surface of pathogens as well as signals emitted by senescent or apoptotic cells (12, 13). Upon activation by these signals, MBL or ficolins undergo conformational changes, and the associated MBL-associated serine proteases (MASP) are activated. Among them, MASP2 cleaves C4 and C2 to form the C3 convertase C4b2a, which is identical to that formed via the classical pathway (14). Pentraxins anchored on pathogen or apoptotic cell surfaces recruit ficolin-2/MASPs to enhance lectin pathway activation, while MBL or ficolin-2 further recruits pentraxins (CRP, PTX3) to engage C1q and amplify the classical pathway (15). Furthermore, MASP3 cleaves pro-factor D to generate active factor D, facilitating the formation of the alternative pathway C3 convertase C3bBb and subsequent C3 cleavage (16, 17). In both the classical and lectin pathways, C3b produced in the previous stage combines with C3 convertase C4b2a to form C5 convertase C4b2a3b. The alternative pathway is continuously active at a low level, and its activation can be facilitated. C3(H2O)Bb cleaves native C3 to generate C3a and C3b (18). Subsequently, factor B, cleaved by factor D, forms the alternative pathway C3 convertase with C3b. The newly generated C3b can combine with the factor B degraded by factor D to form the C3 convertase C3bBb, thus producing a positive feedback effect in the alternative pathway (17, 18). Furthermore, the alternative pathway can be specifically initiated by soluble collectin-12, which recognizes PAMPs and DAMPs and recruits properdin to assemble the alternative pathway C3 convertase (15, 19). In addition, transmembrane collectin-12 interacts with pentraxins (CRP, PTX3) to trigger C1q-dependent classical pathway activation (15, 19). C3b produced in the previous stage of the alternative pathway combines with the C3 convertase C3bBb to form the C5 convertase C3bBb3b. Rather than remaining functionally isolated, these three pathways exhibit mutual cross-activation through coordinated molecular networks composed of pentraxins, collectins, ficolins, and MBL.

The three pathways share convergence points and a common terminal pathway. C5b sequentially binds to C6 and C7. The complex C5b67 exposes membrane-binding sites and non-specifically associates with nearby cell membranes. Subsequently, it binds to C8 and multiple C9 molecules, forming the membrane attack complex (MAC) (20). The insertion of MAC into the cell membrane disrupts the phospholipid bilayer and creates hydrophilic pores. This ultimately leads to a reduction in intracellular osmotic pressure, causing the cells to gradually swell and eventually rupture (20).

### 1.3 Regulation of complement activation

The complement system is tightly regulated by diverse regulatory proteins that safeguard normal cells against inadvertent damage. These regulators are either membrane-bound or present as soluble factors in the circulating plasma (21).

Soluble complement regulators inhibit activation primarily through three mechanisms: decay-accelerating activity (dissociation of convertases), cofactor function, and proteolytic cleavage. Acting as a plasma serine protease inhibitor, C1-INH regulates the classical pathway by accelerating dissociation of C1r and C1s subunits from antigen-antibody-C1 complexes (22). Additionally, it modulates lectin pathway activation through MASP-2 inactivation (23). C4BP inhibits formation of the C3 convertase (C4b2a) in the classical and lectin pathways by binding to C4b (24). Additionally, it serves as a cofactor for Factor I-mediated cleavage of C4b (25). Similarly, Factor H acts as a cofactor for Factor I-mediated cleavage of C3b, thereby inhibiting formation of the alternative pathway C3 convertase (C3bBb) (26).

Cell surface regulatory proteins inhibit complement activation through three principal mechanisms: decay acceleration, cofactor function, and inhibition of C5b-9 formation. CD55 accelerates decay by dissociating C3 convertases of the classical and alternative pathways (27). CD46 and CR1 possess cofactor function, enabling Factor I to cleave C3b and C4b on the cell surface (28, 29). CD59 binds to C8 and C9 to prevent polymerization of the MAC (30).

## 2 The role of the complement system in adaptive immunity

The complement system not only forms the MAC to mediate the killing of pathogens but also serves as a bridge between innate and adaptive immunity. Upon pathogen invasion, the alternative or lectin pathway triggers a cascade reaction by recognizing pathogen surface components. During this process, some active complement fragments can recruit inflammatory cells. Meanwhile, opsonization mediated by complement fragments, such as C3b and C4b, facilitates the uptake and presentation of antigens by antigen-presenting cells (APCs), thereby initiating adaptive immunity. After the production of specific antibodies, the classical pathway is activated to stimulate C3 activation, leading to a more efficient defense.

### 2.1 Humoral immunity

**Figure 2:**
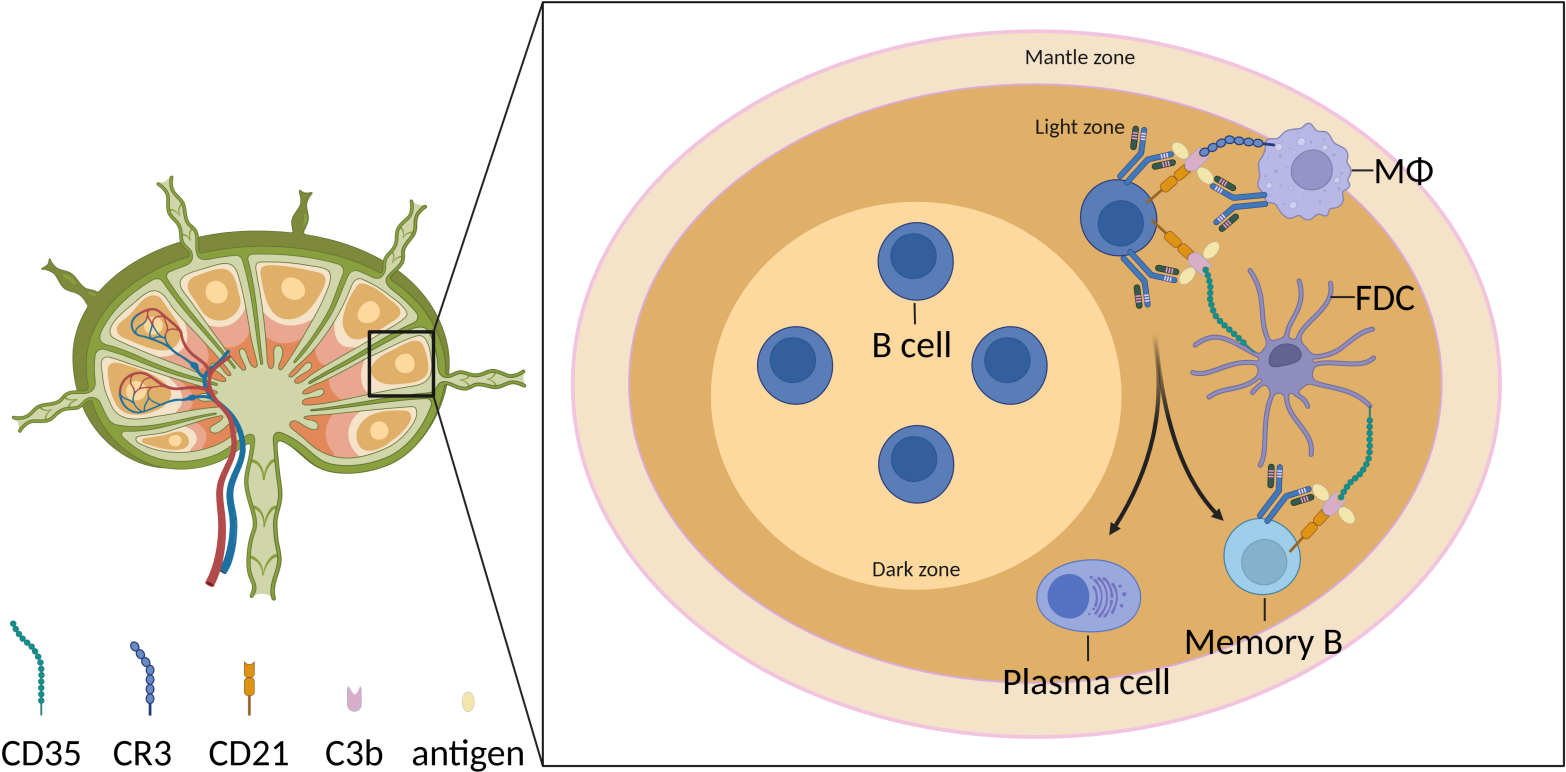
Follicular B cells in the germinal center undergo differentiation mediated by complement-dependent antigen recognition. Complement fragments (illustrated as C3b) form immune complexes with antigen-bound IgG and bind to the surface receptor CR3 on subcapsular sinus macrophages. Marginal zone B cells in the spleen interact with these immune complexes through the complement receptor CD21. Immune complexes are subsequently transferred to follicular dendritic cells (FDCs) via complement receptor-mediated mechanisms.

Relevant studies have confirmed that a transient decrease in circulating complement C3 levels leads to impaired humoral immunity, indicating the involvement of the complement system in the adaptive immune process. Complement receptors CR2 (CD21) and CR1 (CD35) are primarily expressed on B cells and follicular dendritic cells (FDCs), and they bind to complement cleavage products such as C3d, C3dg, and iC3b (**Figure 2**). Research has shown that B cells uptake of antigens coated with C3dg can enhance their signaling (31–34). Related research has confirmed that C3d, C3dg, and iC3b, by binding to CR2, become important costimulatory molecules for B cells, significantly reducing the threshold for signal transduction via the B cell receptor (31, 33, 35). CR2 exerts its function by forming B cell co-receptor complex CR2-CD19-CD81 (36–40). The complement-opsonized antigens bind to CR2, which lowers the threshold for B cell activation and provides essential survival signals (34, 37, 39, 41, 42). Additionally, CR2-mediated antigen-independent signaling is necessary for the survival of B cells within germinal centers (43, 44). Guinamard’s research indicates that TI-II antigens localize to marginal zone B cells (MZB cells) by activating the complement system and binding to complement receptors on these cells (45). C3 and CR1/CR2 are essential for this process, highlighting the significance of complement in both T-dependent and T-independent antigen-antibody responses. FDCs located within the germinal centers of lymphoid follicles play a crucial role in the activation, differentiation, and class-switch recombination of B cells and support the formation and maintenance of germinal centers. The relatively high expression of CR2 and CR1 on FDCs provides an effective mechanism for presenting immune complexes coated with complement. These complexes can subsequently be recognized by B cells, thereby enhancing the reactivity of B cells to antigens (35, 44, 46). On FDCs, CR2 and CR1 serve as the primary receptors for capturing and retaining antigens over the long term, thereby contributing to the formation of memory B cells and the establishment of long-lasting immune memory. Anania found that in wild-type mice, FDCs are predominantly located at the boundary between the light and dark zones of the germinal centers, whereas in Cr2^-/-^ mice, FDCs are more frequently observed outside the germinal centers within the follicles (47). Therefore, the absence of CR2 in FDCs may not be the sole reason for impaired germinal center and antibody responses in Cr2^-/-^ mice. The improper organization of FDCs within the germinal centers may also contribute to the inefficiency of the immune response. Despite the normal expression of both receptors on FDCs, experiments in mice with B cell-specific CR1/CR2 deficiency demonstrated that B cells with complement receptor deficiencies led to a severe impairment in T cell-dependent humoral immunity (48). This indicates that the interaction between CR2 or CR1 expressed on B cells and FDCs with C3 cleavage products is crucial for antigen-specific antibody responses. However, Kovács’ study on human palatine tonsil B cells demonstrated that under non-stimulatory concentrations of anti-IgG/A/M, co-clustering of BCR and CR2 enhanced the Ca^2+^ response but inhibited the expression of activation markers, cytokine production, proliferation, and antibody production (49). Therefore, further investigation is required to determine whether there are significant differences in the genetic background and function of CR2 and CR1 in humans and rodents.

Shivshankar found that vascular endothelial cells stimulated by C3a or C5a significantly upregulated the expression of B cell activation markers FasL, CD69, and IL-R1, which may be associated with the increased secretion of IL-1α, IL-6, and IL-8 by the endothelial cells post-stimulation (50). This suggests that anaphylatoxins may activate B cells through chemotaxis and the involvement of vascular endothelial cells. Cumpelik discovered that germinal center B cells, through Bcl-6, either suppress the expression of decay-accelerating factor or promote the expression of CD59, which allows for the normal cleavage of C3 on the B cell surface and prevents the formation of the MAC (51). Ultimately, C3aR1/C5aR1 signaling activates the key survival pathways mTOR and MYC in B cells, which are required for positive selection (51). In summary, the complement system is crucial for the survival and positive selection of B cells, as well as for the formation, maintenance, and function of germinal centers.

### 2.2 Cellular immunity

The significance of complement proteins lies not only in their ability to enhance immune responses, but also in their role in regulating the type of immune response and influencing the balance among T helper (Th) cell subsets.

#### 2.2.1 The complement system modulates Th cell responses

T cells are regulated by three signals from APCs. APCs present antigens to T cells via MHC class II molecules, inducing their initial activation. The binding of CD80 and CD86 on APCs with CD28 on T cells provides co-stimulation for further activation, while the cytokines produced subsequently promote the proliferation and differentiation of T cells. Cytokines IL-12, IFN-γ, and IL-4 play crucial roles in the polarization of T cells towards Th1 or Th2 phenotypes. TGF-β, IL-6, IL-1β, and IL-23 are involved in cell proliferation and differentiation of Th17 cells. In the absence of pro-inflammatory cytokines such as IL-6, TGF-β promotes the differentiation of naive CD4+ T cells into regulatory T cells (Tregs) (52). Extensive research has indicated that complement components regulate the maturation of APCs and the expression of cytokines in a paracrine or autocrine manner, thereby influencing the three signals required for APCs to activate T cells (53, 54).

**Figure 3:**
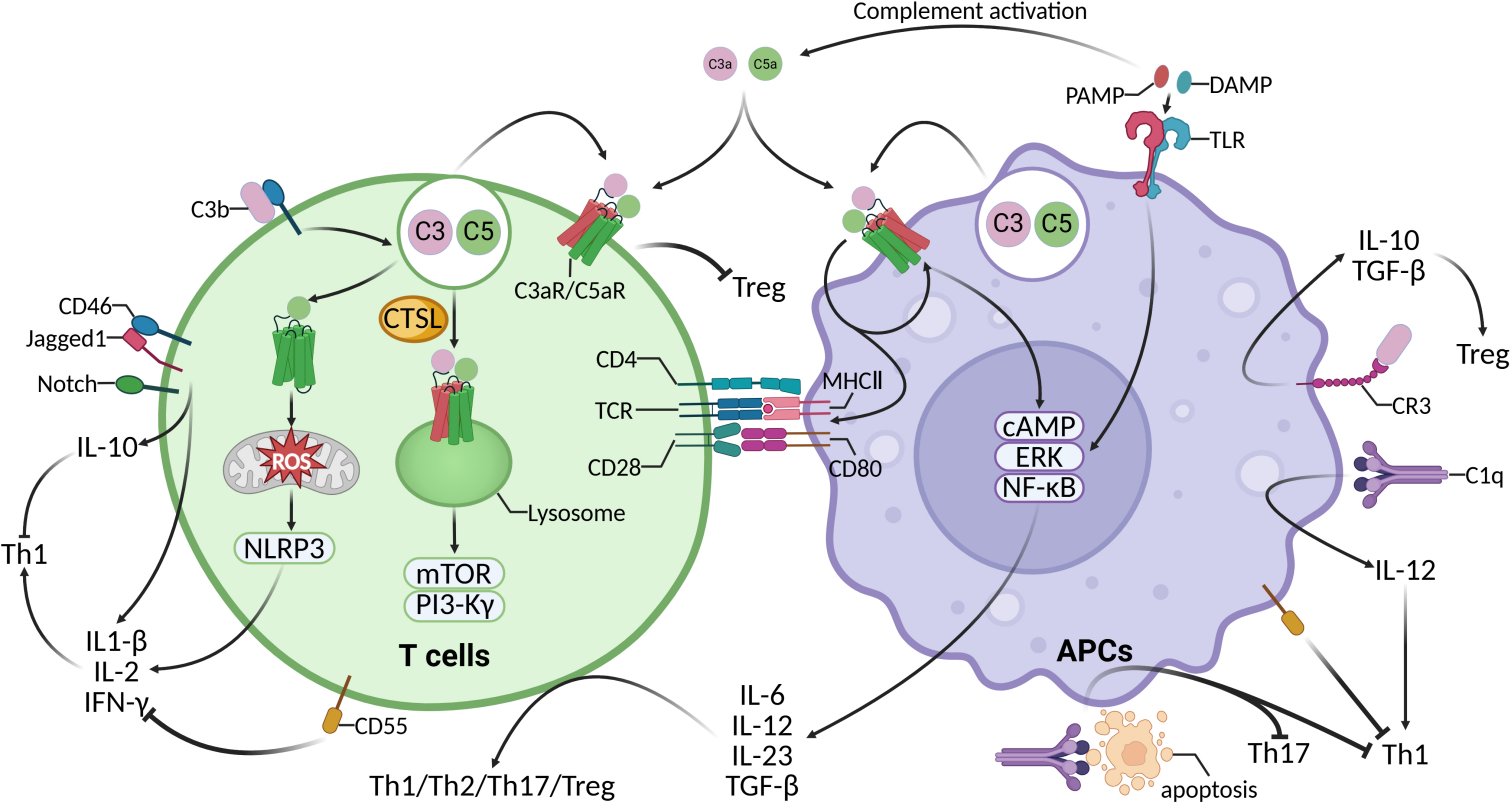
Direct effects of complement signalling activation on T cells and antigen-presenting cells.

During specific interactions between APCs and T cells, C3 and C5 secreted by both cell types are activated by proteases, including C3 convertases and C5 convertases, with concomitant upregulation of C5aR1 and C3aR1 expression (18, 55, 56) (**Figure 3**). C3a and C5a bind to receptors expressed on neighboring cells in an autocrine or paracrine manner, and signals from C3aR1 and C5aR1 crosstalk with Toll-like receptors (TLRs) to induce APCs to activate downstream cAMP, ERKs, and NF-κB (57–62). This, in turn, drives the secretion of cytokines such as IL-6, IL-12, IL-23 and TGF-β, and induces the upregulation of costimulatory molecules such as MHC II, CD80 and CD86 (18, 55, 56, 63, 64). C3aR1 is involved in the TLR4-mediated release of IL-1β and IL-6 in human monocytes and promotes the differentiation of CD4+ T cells towards the Th17 lineage without affecting Th1 and Th2 cytokines (60). C5aR1 signaling in macrophages promotes the production of IL-6 and TGF-β induced by TLR2, TLR4 and TLR9 (65, 66), yet inhibits IL-12 family cytokines (62, 67, 68), which also induces the autoreactive Th17 differentiation of CD4+ T cells. However, C5a and C3a do not inhibit TLR-induced IL-12 production in dendritic cells (DCs), and these anaphylatoxins promote IL-12 production as well as Th1 responses under lipopolysaccharide stimulation (57, 69, 70). Blockade or deletion of C5aR1 in DCs prevents T cell differentiation towards Th1 and enhances the production of TGF-β, IL-6 and IL-23, inducing T cell differentiation into CD25+Foxp3+ Tregs and Th17 (54).

Membrane-type regulatory protein CD55 (Decay Accelerating Factor, DAF) regulates T-cell immunity. Heeger found that the interaction of APCs with T cells was accompanied by rapid production of complement components and downregulation of CD55 expression, and that the absence of CD55 on APCs and T cells enhanced the induction of effector T cells (71). Liu’s study showed that CD55^-/-^ mice exhibit an enhanced Th1 response, which is characterized by increased secretion of IFN-γ and IL-2 and inhibition of IL-10 following antigenic restimulation (72).

#### 2.2.2 Differential effects of complement under different conditions

Activation of TLRs promotes factor B synthesis and activates the alternative pathway, suggesting a feedback mechanism between TLRs and complement-induced responses (73, 74). In the case of complement crosstalk with TLRs, it acts through DCs, which favor Th1 and inhibit Th17, and through macrophages, which favor Th17 and inhibit Th1. The binding of iC3b to CR3 on APCs induces the production of TGF-β and IL-10, which promotes the production of anti-inflammatory phenotype APCs. The interaction between macrophage CR3 and iC3b-encapsulated apoptotic cells inhibits IL-12, and CR3 signaling downregulates TNF production by modulating TLR7/8-induced degradation of MyD88, which is essential for blocking unwanted inflammation and inducing Th1 cell contraction (75–77). However in DCs, CR3 promotes MyD88-dependent TLR4 signaling and assists in the endocytosis of TLR4, promoting T cell proliferation (78). Similarly, apoptotic cell-bound C1q inhibits macrophage and DC-mediated proliferation of Th1 and Th17 T cell subsets (79, 80). However, C1 upregulates the expression of CD83, CD86, HLA-DR, and CCR7 on the surface of DCs, and C1q promotes the production of IL-12 in DCs, which induces the polarization of CD4+ T cells towards Th1 (81). Overall, complement can promote or inhibit Th1 or Th17 differentiation, depending on the APCs involved, antigen type, and environment.

#### 2.2.3 Complement mediates T cell activation and differentiation

Sustained production of C3a and C5a is also necessary for T cell survival. CD4+ T cells are stocked with C3 in endosomes and lysosomes, and their expressed protease, T cell-expressed protease cathepsin L (CTSL), can process C3 in a non-C3-converting enzyme-dependent manner to produce active C3a and C3b (82). Active C3a binds to C3aR1 on lysosomes and aids in T-cell survival by regulating mTOR phosphorylation (82, 83). Liszewski found that CD4+ T cells from C3^-/-^ mice exhibited an attenuated Th1 response, as autocrine CTSL-mediated C3 activation was inhibited, leading to reduced secretion of Th1-associated cytokines IFN-γ and TNF, while exerting a lesser effect on Th2-associated cytokines IL-4 and IL-5 (82). These findings suggest that the activation of C3 induces T cell differentiation. Strainic found that blockade of C3aR1 and C5aR1 signaling on naive CD4+ T cells resulted in termination of PI3-Kγ and mTOR signaling and increased activation of the kinase PKA, culminating in the initiation of auto-induced signaling by TGF-β1 and differentiation of CD4+ T cells into Foxp3+ Tregs (84). Kwan found that signaling through C3aR1 and C5aR1 reduced the inhibitory activity of CD4+CD25hiFoxp3+ Treg cells (85). These findings suggest that C3a and C5a are involved in the induction of Tregs and regulation of cellular functions.

Co-stimulation of CD3 and the complement regulatory protein CD46 induces distinct T cell phenotypes depending on the cytokine milieu. Under low IL-2 conditions, CD3/CD46 co-stimulation promotes Th1 cell differentiation characterized by robust IFN-γ production (86, 87). Conversely, when IL-2 levels are high, CD46 engagement drives a switch from the Th1 phenotype to a regulatory phenotype (Type 1 Regulatory T cell, Tr1), marked by high IL-10 secretion (86, 88–91). This IL-2-dependent transition is mediated by the nuclear translocation of the transcriptional repressor ICER/CREM that attenuates IL-2 production. Furthermore, this process involves signaling through the CD46-CYT1 isoform via the serine-threonine kinase SPAK (86, 92). Meanwhile, CD46 activation links the complement system to T cell immunity through intracellular C5a generation. CD3/CD46 co-stimulation triggers intracellular C5 cleavage, generating C5a, which binds to C5aR1 and drives NLRP3 inflammasome activation in CD4+ T cells (93). This leads to caspase-1-dependent IL-1β secretion, which in an autocrine manner enhances IFN-γ production and promotes Th1 responses (93).

The molecular mechanism underlying CD46-mediated regulation involves crosstalk with the Notch signaling pathway. Jagged1, a canonical Notch ligand, also functions as a physiological ligand for CD46 (94–96). On resting T cells, CD46 sequesters Jagged1, preventing its interaction with Notch receptors and thereby acting as a “molecular brake” on T cell activation (92, 94). Upon TCR activation, CD46 is downregulated, releasing Jagged1 to engage Notch1 and promote Th1 differentiation (94, 96). This spatiotemporal regulation of the CD46-Jagged1-Notch axis is critical for coordinating the initial IFN-γ response and the subsequent transition to IL-10-mediated regulation (94). Thus, CD46 employs distinct mechanisms—ICER/CREM nuclear translocation to attenuate IL-2 production, complement-mediated NLRP3 activation for Th1 induction, and Notch-dependent transcriptional regulation for the Th1-to-Tr1 switch—to temporally regulate T cell responses during different phases of immune activation (86, 93).

Additionally, C5aR2, the second C5a receptor, acts as a negative regulator of C5aR1 and independently regulates T cell differentiation through lipid mediators. C5aR2 sequesters excess C5a and signals via β-arrestins, while forming heterodimers with C5aR1 to promote their internalization and prevent overactivation (97). Activation of C5aR2 orchestrates a shift from prostaglandin E2 (PGE2) dominance to enhanced prostacyclin (PGI2) (98). PGI2 autocrine signaling through PGI2 receptor activates PKA-CREB to induce IL-1R2, a decoy receptor that neutralizes intrinsic IL-1β and licenses IL-10 expression, thereby enabling Th1 cell contraction (98).

## 3 Complement and the gut

Activation of the complement system produces a variety of active fragments with important biological effects involved in immune regulation and inflammatory responses. Activation of the complement system is regulated by various mechanisms, such as soluble and cell membrane surface complement regulatory proteins that control key enzyme activities and MAC assembly. Thus, dysfunction and defects in complement components can lead to complement dysregulation, which in turn leads to the onset and progression of pathological processes (99–103). Enterocytes highly express various complement components that are thought to be essential for the gut epithelium to fulfil its functions (104). The gut not only provides an important physical barrier between the host and the environment, but also is essential for nutrient, electrolyte and water absorption, for inducing tolerance to normal microbiota, and for mounting immune responses against pathogens (105). Complement dysfunction can lead to a variety of diseases, including infections, inflammatory bowel disease and tumors (106, 107). Although the effectiveness and importance of the complement system in the haematological system during immunity to pathogens has been studied in detail, the contribution of the complement system to intestinal barrier function remains unclear.

### 3.1 Intestinal complement under physiological conditions

**Figure 4:**
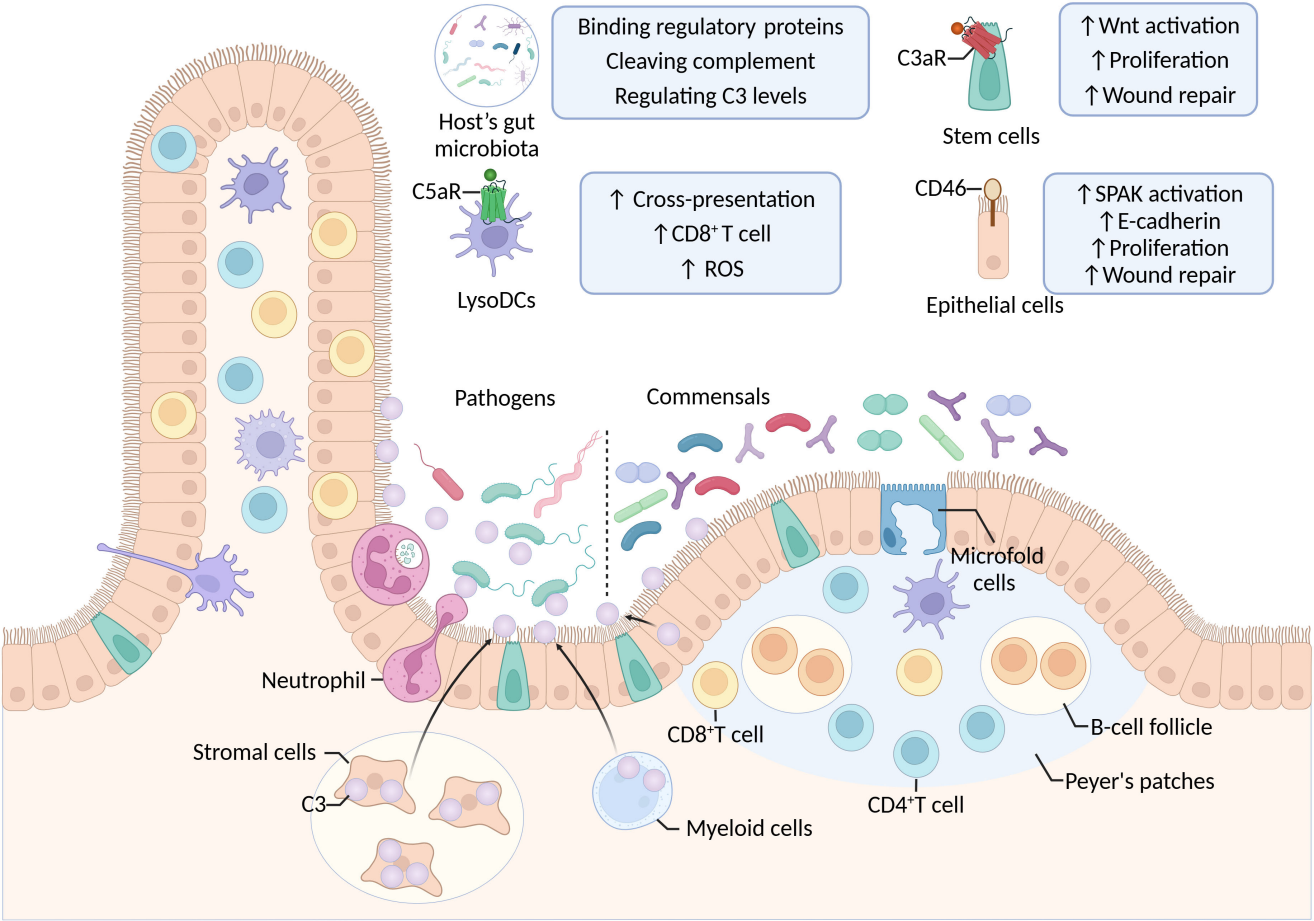
Complement-mediated regulation of gut barrier function and immune cell activity.

The intestine harbors a functionally distinct complement system that operates independently from circulating blood complement. Unlike systemic complement, intestinal complement is selectively expressed to maintain tolerance toward commensal microbiota while preserving rapid pathogen defense capabilities (**Figure 4**).

C3 serves as the central regulator of intestinal immune homeostasis. Under steady-state conditions, intestinal C3 expression is tightly regulated and induced by the local microbiota (108). Studies in both C3-deficient mice and human patients demonstrate that C3 deficiency disrupts intestinal immune balance, shifting cytokine profiles from anti-inflammatory to pro-inflammatory states and increasing systemic IgG responses against commensal bacteria (108). However, the terminal complement components (C5-C9) that form MAC are expressed at minimal levels in the healthy gut, suggesting that intestinal complement primarily functions through opsonization rather than direct lysis (108). Upon mucosal infection, the intestinal complement system undergoes rapid activation. The expression of C3 and alternative pathway factor B becomes significantly upregulated, with intestinal stromal cells serving as the predominant source of local C3 synthesis. This localized production enables neutrophil-mediated phagocytosis through C3b opsonization, effectively clearing invading pathogens (108). Notably, under physiological conditions, limited neutrophil presence in the intestinal lumen prevents excessive complement-mediated clearance of beneficial commensals (108). This spatial restriction ensures that large-scale phagocytosis occurs only during active infection while preserving microbiota stability.

Intestinal complement components also regulate antigen surveillance and adaptive immunity. C5aR1 expressed on microfold cells facilitates mucosal antigen sampling. However, certain pathogens exploit C5aR1-TLR crosstalk (involving TLR1/2 and TLR4) to evade immune clearance (109, 110). In Peyer’s patches, C5aR1 activation on LysoDCs enhances intracellular ROS production, thereby improving antigen cross-presentation and triggering pathogen-specific CD8+ T-cell responses (111, 112). Additionally, IL-22 reinforces mucosal defense by upregulating hepatic C3 synthesis and promoting C3 deposition on bacterial surfaces (73, 113).

Beyond immune defense, complement signaling maintains intestinal barrier integrity and tissue regeneration. The complement regulatory protein CD46 stimulates intestinal epithelial cell proliferation and accelerates wound healing through direct interaction between the cytoplasmic kinase SPAK and the transmembrane adhesion molecule E-cadherin (114). Concurrently, C3a-C3aR1 signaling activates Wnt pathways, upregulating intestinal stem cell markers (Lgr5+) and proliferation markers (Ki67). This axis promotes epithelial regeneration following mucosal injury, highlighting the dual role of intestinal complement in both immune surveillance and tissue homeostasis (115).

### 3.2 Immune evasion by pathogens

While the gut microbiome has been extensively studied in terms of its composition and function, research on how specific mucosal-adherent bacteria interact with the complement system at the mucosal interface remains limited. Since these mucosa-associated bacteria are in close contact with intestinal epithelial cells, they constitute an important group that is thought to play a major role in inflammation in patients with IBD (116). The three complement pathways centered on C3 achieve lysis of gram-negative bacteria by driving the formation of MAC or by promoting phagocytosis and killing by neutrophils and macrophages. In contrast, pathogens impair complement-mediated recognition and bacterial killing by inhibiting the complement cascade.

#### 3.2.1 Binding to complement regulatory proteins

Some pathogenic bacteria have surface proteins that confer resistance to complement, such as Rck, TraT, PagC, Ail, which can recruit complement regulatory proteins to inhibit the formation of C3 convertase, thereby hindering the complement cascade and the formation of the MAC. Ail is a 17 kDa *Yersinia enterocolitica* outer membrane protein that mediates adhesion to and invasion of intestinal epithelial cells. It was found that the *Y. enterocolitica* serum resistance protein Ail binds the complement regulatory protein C4b to evade complement-mediated clearance in the human host (117). Derek K. Ho found that Rck expressed in *Escherichia coli* binds factor H in human serum to inhibit alternative pathway-mediated C3b and MAC deposition, and this study demonstrates that factor H binding to Rck promotes factor I-mediated C3b cleavage (118). The team also found that Rck recruits and binds C4BP to inhibit C3b and MAC deposition mediated by the classical or lectin pathways (119). The gene pagC, activated by the two-component system PhoP-PhoQ in *Salmonella enterica*, has a significant effect on the formation of bacterial outer membrane vesicles (OMVs). OMVs produced by PagC-expressing bacteria can bind complement component C3b in a dose-dependent manner and recruit factor H to inactivate it (120). Homologous proteins Ail, Rck, and PagC, with high sequence similarity in transmembrane structural domains, may have structures particularly suited for recruiting complement regulatory proteins, which results in inefficient recognition of pathogens by complement receptors on phagocytes and impaired complement-mediated opsonization (121, 122). Inhibition of key nodes in the complement pathway increases the chances of pathogens escaping complement attack, and increasing evidence also suggests that these pathogens have the ability to bind multiple complement regulatory proteins. Among the various complement-avoidance mechanisms, the recruitment of complement regulatory proteins is by far the most widely used strategy for escaping complement attack (123).

#### 3.2.2 Cleavage of complement components

Pathogen proteases, such as the serine protease Pic produced by *Shigella fowleri* and Shiga toxin-producing *E. coli*, can directly cleave complement components to block the complement cascade. Specifically, Pic cleaves C2 and C4, components of the classical and lectin pathways, and synergizes with factors I and H (124). The surface protease PgtE from *Salmonella* is able to cleave the C3b, C4b, and C5 components of the complement system (125). Additionally, the toxin PET from enteroaggregative *E. coli* can cleave C3, C5, and C9 components, thereby inhibiting the formation of the MAC (126). Sorbara discovered that C3-encapsulated *Listeria monocytogenes* is targeted by autophagy through direct C3/ATG16L1 interactions, resulting in autophagy-dependent growth restriction, while some Enterobacteriaceae such as *Shigella* and *Salmonella* can escape from C3-dependent growth restriction via shedding C3 mediated by the proteases IcsP and PgtE (127).

The human gut is colonized by a large number of different microorganisms, and this rich and diverse microbial ecosystem plays a crucial role in maintaining epithelial barrier integrity. Research has shown that intestinal dysbiosis is closely associated with intestinal inflammation, and alterations in the composition of gut microbiota may lead to impaired physiological functions and are associated with a range of human diseases, such as metabolic disorders, inflammatory diseases, and tumorigenesis (128–130). Understanding the complex interactions between the gut microbiota and mucosal immune system could potentially lead to the development of novel therapeutic strategies for treating gut-related diseases.

### 3.3 Inflammatory diseases of the intestine

#### 3.3.1 Inflammatory bowel disease

As a group of chronic, recurrent inflammatory diseases of the intestine, IBD is divided into two well-defined subtypes: Crohn’s disease (CD) and Ulcerative Colitis (UC). UC is confined to the colon and presents with severe mucosal inflammation and submucosal ulcers. In contrast, CD is characterized by discontinuous transmural inflammation that can affect all layers of the gastrointestinal tract (131). Continued activation and imbalance in the regulation of the intestinal complement system disrupts the barrier function of the gut, leading to severe intestinal inflammation in patients with IBD (132–136). Deposits of MAC and immune complexes in the colonic submucosal vessel wall of patients with IBD suggest that local complement activation occurs in intestinal lesions (137–139). Enhanced expression of C3 and IL-17 in the inflamed mucosa of patients has been found (140), and the TLR4-C3 axis can control the intestinal immune response during chronic colitis (141), suggesting a correlation between the complement system and the pathogenesis of IBD. In lesions of active UC, complement activation is often observed along the brush border of surface epithelial cells, which correlates with the presence of IgG1 autoantibodies (142). In patients with UC, hTM5-specific IgG autoantibodies present in the serum promote C3b deposition and destruction of colonic epithelial cells, indicating a potential direct pathogenic role (143). Autoantibodies trigger complement attack on the brush border antigens of colonic epithelial cells, leading to their lysis (142). In CD, epithelial deposition of C3b and MAC in the absence of IgG and classical components (C1q and C4c) has been reported (144). Factor B is significantly overexpressed in the intestinal mucosa of patients with CD, where its biosynthesis is regulated by TLR3 (145). Moreover, genetic coding variants in Factor B are associated with increased susceptibility to perianal CD (146). These findings suggest that alternative pathway activation is more prominent in CD.

#### 3.3.2 Colitis

Mice lacking C3 or factor B were protected against acute DSS-induced colitis, whereas those deficient in C1q and MBL displayed increased severity of colonic inflammation (147, 148). Targeted inhibition of the complement system using CR2-Crry, which blocks all pathways, or CR2-fH, which targets the alternative pathway, led to a significant increase in the anti-inflammatory cytokine IL-10 (147). Furthermore, deletion of C3aR1 or C5aR1 significantly reduced the levels of pro-inflammatory cytokines (149–151). In the DSS-induced chronic colitis model, mice with deficiencies in complement proteins C1q, MBL, or C3 showed heightened mucosal damage and increased susceptibility to infections, whereas mice lacking factor B showed mitigated mucosal damage (152). In contrast, administration of CR2-fH attenuated the inflammatory response and fibrosis, while also reducing the infiltration of intestinal B cells, macrophages, and DCs (152). In chronic colitis, C5aR1^-/-^ mice showed severe tissue damage, increased granulocyte infiltration, and lower IL-4 mRNA levels, suggesting that C5aR1 plays a role in modulating the polarization of Th cells towards a Th2 phenotype (150). This highlights the distinct roles that C5aR1 plays in acute versus chronic colitis, indicating that its function may vary depending on the disease phase. Inhibition of the C3a-C3aR1 axis during different stages of colitis can modulate the expression of caspase-11 and inflammatory factors, which in turn influences the disease prognosis (153).

The above suggests that the classical or lectin pathway has a role in protecting the mucosa from injury and infection, whereas the alternative pathway is closely related to the pathogenesis of colitis and the immune response, and that complement may have a dual role at different stages of inflammation.

### 3.4 Complement and intestinal tumors

**Figure 5:**
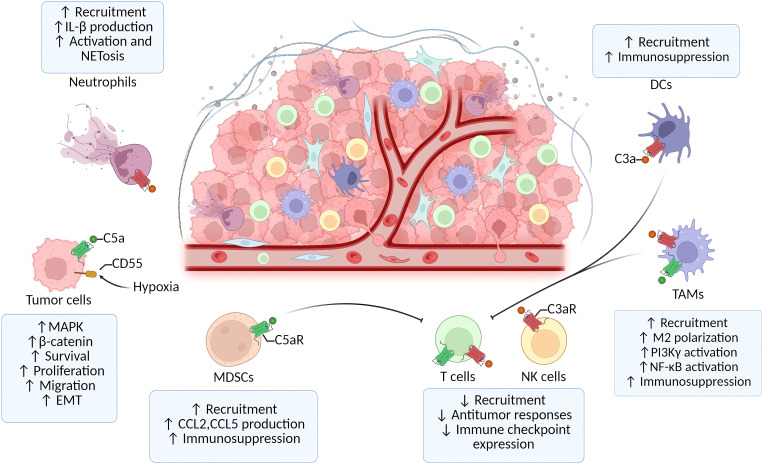
Complement system-driven immune modulation in tumor growth and metastasis.

The TME plays a key role in cancer development, progression, metastasis, recurrence, and drug resistance, with cancer cells, stromal cells such as immune cells and fibroblasts, and extracellular components constituting the TME (154–156). Tumor-associated macrophages (TAMs), tumor-associated neutrophils (TANs), and myeloid-derived suppressor cells (MDSCs) are the primary immunosuppressive cell types that infiltrate the TME (157). Additionally, tumor proliferation and invasion are associated with DCs, cancer-associated fibroblasts (CAFs), and Tregs (158–160). The TME is rich in complement proteins, and there is evidence suggesting that these components may be pro-tumorigenic and immunosuppressive within the TME (161, 162). As both tumor cells and stromal cells produce abnormal complement proteins, the abnormal activation of the complement system in the TME promotes tumor development (**Figure 5**). This occurs by inhibiting inflammation and stromal cell immunity, as well as by enhancing tumor cell proliferation, epithelial-mesenchymal transition (EMT), and migratory spread (161–163).

#### 3.4.1 Complement and complement receptors in TME

Serum C3a concentrations were found to be increased in patients with colon cancer, and high levels of C3 were associated with poor prognosis in colorectal cancer as well as with levels of immune cell infiltration (164, 165). Inhibiting C3 levels can potentially suppress tumor progression by enhancing effective immune responses against tumors, increasing sensitivity to radiotherapy, and reducing the immunosuppressive effects of the TME (166–168).

Studies using chimeric mouse models suggest that the pathogenesis of colitis-associated colorectal cancer is linked to C3 originating from myeloid cells rather than non-myeloid cells (169). In small bowel tumors, complement activation leads to C3a-dependent neutrophil activation and the formation of neutrophil extracellular traps (170). This process results in a hypercoagulable state of the blood, which contributes to the metastatic spread of the tumor.

High C3ar1 expression in patients with colorectal cancer contributes to the production of an immunosuppressive tumor microenvironment by maintaining NK cells, macrophages, neutrophils, and DCs in a resting state (171). Mechanistically, tumor cell-derived C3a drives M2 macrophage polarization via the C3a-C3aR1-PI3Kγ signaling axis, thereby suppressing anti-tumor immunity (172). Adhesion and migration of NK cells are regulated by lymphocyte function-associated antigen-1 (LFA-1). Physical interaction between C3aR1 and LFA-1 on NK cells maintains LFA-1 in a high-affinity conformation, thereby inhibiting NK cell infiltration into tumors (173). These findings suggest that the C3a-C3aR1 axis can suppress CD8+ T cell responses and prevent NK-cell infiltration, thereby creating an immunosuppressive tumor microenvironment. In the C3aR1^-/-^ mouse model, the absence of C3aR1 in the colon activates a microbiota-mediated inflammatory response. This response results in the TME with a pronounced immune infiltrate characterized by Th1, Th17, and cytotoxic T cells (171). This shift promotes the transition from a cold to a hot tumor, making it more susceptible to immune checkpoint-blocking therapy (171).

C5aR1 on myeloid cells is essential for colorectal tumorigenesis. The recruitment of MDSCs through C5a-C5aR1 signaling contributes to the suppression of CD8+ T cells and can modulate the local production of a variety of pro- and anti-inflammatory cytokines and chemokines, resulting in a pro-tumor immunosuppressive microenvironment (174). TAMs also express C5aR1, and their expression of C5aR1 is negatively correlated with the degree of tumor differentiation. Related studies have found that C5a can mediate the polarization of macrophages to the M2 type and promote tumor metastasis to the liver through the NF-κB pathway (175). C5 can be cleaved in a convertase-independent manner in colonic epithelial cells, in addition to its cleavage by myeloid cells. Aberrant activation of β-catenin is an important factor in colorectal cancer. C5a-C5aR1 signaling triggers the assembly of intracellular C5aR1 with KCTD5/cullin3/Roc-1 and β-catenin complexes. This interaction promotes the polyubiquitination of β-catenin from K48 to K63, thereby facilitating colorectal tumorigenesis (176).

#### 3.4.2 Complement regulatory proteins in TME

Studies have shown that CD46, CD55, and CD59 are up-regulated in colorectal cancer. In particular, the expression levels of CD55 and CD59 correlate with the degree of differentiation and the TNM (Tumor-Node-Metastasis) stage of the cancer (177). Additionally, CD46 is also significantly overexpressed in colorectal cancer tissues and cell lines, and its expression is positively associated with tumor invasion and advanced stages (178). CD46, CD55, and CD59 have been identified as potential biomarkers for poor prognosis in colorectal cancer patients (165, 178–180).

It has been found that knocking down CD55 induces apoptosis in tumor cells, thereby reducing the growth of colon cancer in mice (181). Additionally, the expression of CD55 is associated with the induction of a hypoxic environment and regulated by the STAT3/STAT6/p38MAPK signaling pathway (178, 181). Similarly, CD46 transcription is directly controlled by STAT3/STAT6/p38 MAPK signaling in colon cancer cells (178). Knockdown of CD46 or CD59 alone significantly triggers caspase-dependent apoptosis, increases MAC deposition, and suppresses colorectal cancer growth in mice (178, 182). Moreover, CD59 enables tumor cells to evade complement-dependent cytotoxicity by blocking the assembly of the terminal complement complex, and its high expression is associated with shorter disease-specific survival (180).

Recent studies have revealed the emergence of new B-cell subpopulations in patients with tumors after chemotherapy (183). These subpopulations activate T-cell-associated anti-tumor immunity via ICOSL, a process regulated by the complement regulatory protein CD55. CD55 expression was negatively correlated with the number of ICOSL+ B cells. Targeting CD55 promotes the production of ICOSL+ B cells, which in turn enhances anti-tumor immunity after chemotherapy (183). Furthermore, silencing CD46 or CD59 markedly sensitizes colon cancer cells to chemotherapeutic agents such as doxorubicin, providing a rational combination strategy for chemotherapy (178, 182). Thus, targeting complement regulatory proteins (CD46, CD55, CD59) may serve as an innovative strategy to improve the efficacy of chemotherapy-based treatments.

The role of the complement system in tumors is complex and multifaceted. It can either act as part of tumor immune surveillance or promote tumor immune escape and progression. Therefore, an in-depth study of the mechanisms by which the complement system influences tumors is crucial for developing new strategies in tumor immunotherapy.

## 4 Conclusion

The complement system plays a multifaceted role in intestinal health and disease. It is involved not only in the defense response against pathogens but also in regulating the gut immune response and inflammatory processes. Disorders of the complement system are associated with the development of IBD and intestinal tumors, making them potential therapeutic targets. Future research should further explore the specific mechanisms by which the complement system contributes to intestinal diseases and how these diseases can be treated by modulating the complement system. With a deeper understanding of the role of the complement system in the gut, we can look forward to developing novel therapeutic strategies to improve the prognosis of intestinal diseases and enhance patients’ quality of life.
